# Supplementary material for: Dietary Thymol Supplementation Promotes Antioxidant Responses and Thermal Stress Resistance in Rainbow Trout, Oncorhynchus mykiss
Source: Animals (Basel). 2024 Oct 16;14(20):2988. doi: 10.3390/ani14202988 (PMC11503809; doi:10.3390/ani14202988)
Supplement: Supplementary file 1 [file animals-14-02988-s001.zip › animals-3231304-supplementary.pdf]

Table S1: Growth performance data

Dietary thymol	Initial weight	initial biomass	final weight	final biomass	weight gain %	SGR	feed intake	Feed efficiency
0	5.4	108	25.17829	503.5658	366.2646	2.749256	440.5887	0.897812
0	5.5	110	24.52654	490.5308	345.9371	2.669657	431.6595	0.881553
0	5.7	114	24.28253	485.6506	326.0093	2.58802	422.8371	0.878945
50	5.5	110	25.90457	518.0913	370.9921	2.76727	454.2259	0.898432
50	5.6	112	26.1994	523.9881	367.8465	2.755304	464.1544	0.88761
50	5.7	114	24.91186	498.2372	337.0501	2.63371	433.1965	0.886981
100	5.4	108	24.64715	492.943	356.4287	2.711183	438.9737	0.876916
100	5.5	110	26.84052	536.8104	388.0095	2.830651	486.0863	0.878055
100	5.6	112	27.02098	540.4196	382.5175	2.810441	473.4947	0.904803
200	5.4	108	24.61356	492.2712	355.8067	2.708748	431.7449	0.890042
200	5.5	110	23.99906	479.9812	336.3465	2.630833	415.0666	0.891378
200	5.6	112	24.06361	481.2723	329.7074	2.603454	426.6902	0.865434
400	5.3	106	24.06219	481.2437	354.0035	2.701669	409.5902	0.916144
400	5.6	112	26.71523	534.3046	377.0576	2.79012	457.8819	0.9223
400	5.7	114	25.61034	512.2068	349.3042	2.683089	446.6187	0.891603
800	5.5	110	24.11597	482.3195	338.4722	2.639511	424.6585	0.87675
800	5.4	108	23.05719	461.1438	326.985	2.592105	397.4777	0.888462
800	5.7	114	25.57951	511.5902	348.7633	2.680938	440.0128	0.903588

Table S2: Immunological data

	lysozyme	ACH50	Total Ig	total protein	albumin	globulin
0	24	32	5.2	3.4	1.52	1.88
0	29	41	6.3	3	1.5	1.5
0	31	28	7.1	3.8	1.82	1.98
50	25	29	6.5	2.9	1.46	1.44
50	28	35	8.9	3.4	1.7	1.7
50	33	44	7.4	3.8	1.82	1.98
100	31	36	7.1	3.6	1.74	1.86
100	27	48	9.2	3.7	1.7	2
100	40	40	6.3	3	1.5	1.5
200	25	37	5.9	3.7	1.78	1.92
200	30	30	6	3.3	1.6	1.7
200	29	40	7.4	3.1	1.54	1.56
400	24	42	6.5	3.2	1.58	1.62
400	29	31	5.9	3	1.55	1.45

400	26	36	8.4	3.6	1.74	1.86
800	27	32	7.1	3.5	1.7	1.8
800	23	39	6	3.1	1.6	1.5
800	29	41	5.7	3	1.5	1.5

Table S3: Antioxidant and stress data

	dietary thymol	MDA	SOD	CAT	GPx	Gr	GSH	Ascorb	TAC	glucose	cortisol
before stress	0	75	32	54	51	46	2.5	2	2.56	41	15
before stress	0	90	41	69	38	61	1.6	1.51	4.25	43	19
before stress	0	70	36	41	65	50	1.3	1.62	3.25	46	24
before stress	50	50	33	56	41	55	3.2	2.11	3.56	43	18
before stress	50	75	36	59	67	76	1.8	1.98	5.61	40	24
before stress	50	80	41	71	55	90	1.4	2.19	6.25	47	20
before stress	100	40	48	88	98	75	3.6	2.55	7.23	49	16
before stress	100	29.5	71	59	75	121	4.6	3.01	12.5	43	12
before stress	100	35	66	77	115	100	5.1	2.39	6.89	53	20
before stress	200	40	44	58	74	100	2.3	2	6.56	41	26
before stress	200	48	58	69	89	69	4.2	1.68	8.12	49	20
before stress	200	65	41	75	100	85	3.1	2.1	9.68	45	14
before stress	400	75	42	71	61	61	3.7	2.01	1.36	42	17
before stress	400	60	55	60	75	55	4.3	1.69	2.91	40	29
before stress	400	100	60	64	50	42	3	1.76	4.12	35	21
before stress	800	90	42	65	49	43	1.6	1.15	2.39	43	21
before stress	800	110	46	71	31	59	2.8	1.78	3.11	35	17
before stress	800	70	51	58	42	63	2.2	1.81	4.29	39	24

after stress	0	155	60	97.2	145	78.2	0.7	0.48	1.28	85	65
after stress	0	171	82	115	110	96	0.8	0.95	2.125	70	47
after stress	0	142	70	79	104	85	0.9	0.78	1.625	76	79
after stress	50	92	60	100.8	73	110	1.6	0.91	1.78	76	46
after stress	50	135	59	110	100	99	0.8	1.16	2.805	81	67
after stress	50	140	66	96	121	138	1	1.22	1.36	69	41
after stress	100	60	49	75	121	200	2.5	1.78	4.3	56	39
after stress	100	49	54	61	100	170	2.3	2.01	6.25	67	54
after stress	100	55	69	83	89	136	1.9	2.41	5	69	27
after stress	200	54	49	130	160	119	1	1.78	3.28	55	47
after stress	200	69	62	120	120	160	1.9	1.65	2.3	60	39
after stress	200	85	64	110	172	136	2	1.26	2.9	68	61
after stress	400	120	84	109	100	79	1.7	1.11	1.1	71	45
after stress	400	102	106	116	140	98	1.6	1.09	1.2	60	62
after stress	400	160	111	96	110	116	2	0.91	2.06	50	55
after stress	800	172	84	125	102	110	0.8	0.81	1.195	56	66
after stress	800	180	100	111	75	105	0.6	0.7	1.555	59	57
after stress	800	133	109	100	96	79	1.1	0.59	1.09	75	50

Table S4: Survival data

dietary thymol	total fish number	dead fish	survival %
0	10	3	30
0	10	4	40
0	10	5	50
50	10	7	70
50	10	6	60
50	10	5	50
100	10	7	70

100	10	6	60
100	10	8	80
200	10	7	70
200	10	7	70
200	10	6	60
400	10	7	70
400	10	6	60
400	10	5	50
800	10	6	60
800	10	5	50
800	10	6	60
